# “Condoms don't cross your mind when you're hungry”: Challenges to safe sex in Libyan IDP camps; A qualitative study

**DOI:** 10.1016/j.puhip.2026.100722

**Published:** 2026-01-11

**Authors:** Fauzi Elamouri, Amera Muftah A Alamori, Jürgen Kurt Rockstroh

**Affiliations:** a College of Public Health Sciences, Chulalongkorn University, Bangkok, Thailand; b Medical School Hamburg, Hamburg, Germany; c Department of Medicine I, University Hospital Bonn, Bonn, Germany; d Department of Pediatrics, University Hospital Tripoli, Tripoli, Libya

**Keywords:** HIV, Condom, Sexual health, Internally displaced persons, Libya

## Abstract

**Objectives:**

This qualitative study among internally displaced heterosexual men in post-conflict Libya investigates the attitudes, experiences, and barriers related to condom use within this vulnerable population.

**Study design:**

Qualitative study using semi-structured, face-to-face in-depth interviews.

**Methods:**

Semi-structured, face-to-face in-depth interviews were conducted with 21 internally displaced (IDPs) male participants, aged 18–35. Participants were selected through purposive sampling, focusing on their displacement status and willingness to discuss sexual health practices. The interviews explored knowledge, experiences, and challenges related to condom use using a guided protocol. Braun and Clarke's thematic analysis framework was used to analyse the data.

**Results:**

A total of 21 male heterosexual IDPs, aged 18–35, residing in IDP camps in Tripoli were recruited for the study. Only 9.5 % (n = 2) ever used a condom. Key barriers to condom use included financial constraints, geographic inaccessibility, cultural taboos, and stigma. Risk factors for condomless sex included lower knowledge about HIV and STIs, misconceptions about condom efficacy, lack of sexual health education, fear of social judgment, and negative perceptions about reduced pleasure. The theme of basic needs overriding safer sex practices was highlighted.

**Conclusions:**

This study reveals significant gaps in sexual health knowledge and condom use among young displaced men in Libya. Targeted, culturally sensitive interventions are urgently needed to improve sexual health education, promote positive attitudes towards condom use, and enhance access to contraceptives, particularly in post-conflict settings.

## 1 Introduction

Since the removal of Muammar Gaddafi in 2011, Libya has been engulfed in a state of turmoil. The attempt to overthrow his regime did not succeed in establishing stable political structures. Instead, Libya has been overwhelmed with political chaos, amidst rival governments and military groups struggling for control over different regions of the country. This fragmentation has resulted in a weakened central government, unable to provide consistent public services, including healthcare [1]. The ongoing conflict has created a population of Internally Displaced Persons (IDPs) who are individuals which were forced to flee their homes, but who remained within their country's borders with 125,802 people displaced in Libya in 2023.These IDPs are facing challenges in accessing basic healthcare services, including those related to sexual and reproductive health [2].

Libya has historically had a low reported prevalence of HIV, which however is likely to be impacted by underreporting and HIV-associated stigma [3]. Over the last years a steady increase in HIV prevalence from 0.13 % in 2004 to 0.2 % in 2019 and 2022 has been reported for Libya [4]. Interestingly, internal displacement has been discussed as one factor contributing to the spread of HIV [5]. The main transmission routes for HIV in Libya include heterosexual contacts, with additional cases attributed to unsafe medical practices, such as the reuse of needles, and increasingly intravenous drug use [6,7]. The conflict has disrupted HIV monitoring and treatment programs, making it difficult to assess the current situation accurately.

Libya's population is predominantly young, with more than 50 % under the age of 30 [8]. This demographic is particularly vulnerable to the effects of the ongoing conflict, including disruptions in education and health services. The lack of comprehensive sex education has left many young people without the necessary knowledge to protect themselves from STIs, including HIV [9]. Condom use remains low in Libya, largely due to cultural and religious taboos. Discussions around sexual health are often considered inappropriate, and unmarried individuals, in particular, face significant social stigma if they are found purchasing or using condoms [10,11]. Studies in similar post-conflict settings have shown that barriers like poverty, displacement, and a lack of privacy consistently hinder safe sex practices among men, prioritizing basic needs over preventative measures [12,13]. Furthermore, there are few, if any, public policies promoting condom use, and free distribution programs are nearly nonexistent, especially in IDP camps and conflict zones. But condom use in heterosexual men also remains low in other studies outside of IDPs for a whole number of different reasons such as condoms decreasing pleasure and condom associated erection problems, alcohol or drug use prior to sex, anal sex, not carrying a condom, low education, shame about buying condoms, and lack of sexual health education altogether [[14], [15], [16]].

Libya is a predominantly Muslim country where cultural and religious norms strongly influence attitudes toward sexual health and contraception. Many Libyans adhere to conservative interpretations of Islam, which emphasize premarital chastity and marital fidelity, creating resistance to condom use and discussions about sexual health [17]. The World Health Organization (WHO) advocates for the integration of sexual and reproductive health (SRH) services into broader humanitarian aid programs in conflict-affected regions [18], yet tailored, local data on barriers in Libya remains scarce.

Given the strong cultural and religious norms surrounding sex, and the sensitivity of the topic, focusing on heterosexual men is a critical public health necessity for several reasons. First, men are widely considered the primary decision-makers regarding condom use in this cultural context. Second, while the reported HIV rate is low, the destabilizing effect of conflict, disruption of education, and increased displacement-related risk behaviors mean this population is highly vulnerable. Indeed, targeting this demographic provides essential data for informing behavioral change interventions.

In summary, the ongoing conflict in Libya has led to a collapse of the national healthcare system, leaving many areas without access to basic services, including those related to sexual and reproductive health. IDPs, in particular, are living in overcrowded and unsanitary conditions, which increases the risk of HIV and other STIs. Libya currently lacks a coherent national policy on condom use and HIV prevention. There are no widespread public health campaigns or free distribution programs to promote condom use, even in areas most vulnerable to HIV and other STIs. Therefore, the aim of this study was to investigate the attitudes, experiences, and barriers related to condom use within male IDPs to better understand the sexual health behaviors in post-conflict Libya and to find evidence for the need of sexual health education interventions. This investigation considers the contextual complexity of sexual risk, which is highly modulated by relational dynamics and individual reproductive intent.

## 2 Methods

### 2.1 Study design and setting

This study employed a descriptive qualitative design utilizing thematic analysis to deeply explore the attitudes, experiences, and barriers related to condom use among young internally displaced heterosexual men in post-conflict Libya. The research was conducted within Internally Displaced Persons (IDP) camps located in Tripoli, Libya, a setting characterized by political instability and disruptions to public services [1].

### 2.2 Recruitment and data collection

To reach potential participants, announcements were made in camp mosques with the assistance of the Imam and community focal points. Additionally, eligible participants were invited during visits to common areas such as dining rooms and activity spaces. To ensure privacy and comfort, interviews were conducted in locations chosen by the participants, such as private halls or other quiet areas within or outside the camp. The participants received a three Gigabyte internet voucher by the end of the interview as a sign of appreciation.

In August 2023 (recruitment took place between August 1st and 31st 2023), we conducted in-depth interviews with 21 (IDPs), who all identified as heterosexual men. Previous research has shown that thematic saturation could be reached with sample size ranging from 5 to 25 participants [19], and interviews continued until thematic saturation was achieved, meaning no new major themes or concepts emerged from the final interviews.

The interviews focused on several key topics related to condom use and frequency of condomless sex activities. These included [1]: perceptions and attitudes of IDP men towards condom use in the post-conflict context [2], personal experiences that influenced their use or non-use of condoms [3], the personal, social, and economic factors affecting their condom use [4], their knowledge of condom use as a preventive measure against HIV and other STIs, and [5] the barriers they perceived in accessing and using condoms.

Interviewers received comprehensive training, including protocols for conducting qualitative interviews and strategies for ensuring consistency and adherence to research standards. All interviews were conducted in Arabic, ensuring strict confidentiality by holding them in private rooms within the IDP residences. Due to heightened participants' concerns about stigma and being recognized in this sensitive setting, the interviews were not audio-recorded; instead, detailed written interview summaries were created immediately after each session. The notes captured direct quotes, core concept, and observations of non-verbal cues such as facial expressions. Participants were verbally presented with a summary of their key points at the end of the interview to verify accuracy. Each interview was followed by a debriefing session lasting between 10 and 20 min, conducted by the research team members. These sessions aimed to identify emerging themes and monitor the progression toward data saturation**.** The interviews, averaging 45 min in duration, provided valuable insights into the factors influencing condom use among young IDP men in Libya.

This study received ethical approval from the Review Committee for Research Involving Human Research Subjects, Health Science Group, Chulalongkorn University, Bangkok, Thailand (COA No. 166/66). Additional permission was obtained from local government authorities and IDP camp management to conduct the research. Participants were informed about the study through written participant information sheets, which provided details about the study's purpose, benefits, data collection process, and their rights. Since all participants were literate, they could read and understand the provided information without concern. They were given the opportunity to ask questions before deciding whether to participate.

Written consent was not obtained to protect participants' privacy and avoid potential stigma. Instead, informed consent was obtained orally from all participants. Confidentiality was maintained by securely storing all written interview summaries, transcriptions, and related documents in a locked location accessible only to authorized researchers. No identifying information was included in the analysis or final report. All research records will be securely stored for five years and then deleted in compliance with ethical guidelines.

### 2.3 Data analysis

The **written interview summaries** were translated into English by an official translation office with medical expertise. We conducted a thematic analysis following Braun and Clarke's [20] six-phase framework to systematically examine the data.

To familiarize ourselves with the data, we carefully read the translated **written interview summaries** transcripts multiple times, noting key observations, including both verbal and non-verbal cues. In the initial coding phase, two investigators (FE, JR) independently identified and coded meaningful features relevant to the research question. Coding discrepancies were resolved through team discussions, ensuring consistency and accuracy. These codes were then grouped into broader themes based on conceptual similarities.

Next, we refined and reviewed these themes to ensure they accurately reflected the dataset. We further refined the themes by comparing them against the coded data and study objectives, assigning clear and descriptive labels. Finally, we developed a thematic diagram to illustrate relationships between themes and ensure a comprehensive, flexible analytical framework.

This approach allowed us to systematically identify and interpret key patterns within the data, ensuring that our findings were grounded in the participants’ narratives and the research context.

## 3 Results

### 3.1 Characteristics of participants

Twenty-one male participants (N = 21) were interviewed. All participants were recruited from internally displaced persons (IDP) camps in Tripoli. Participants ranged in age from 18 to 35 years (M = 25. SD = 4.2). The sample was predominantly single (n = 18.85 %). Regarding occupation, five participants (n = 5.23 %) were students, six (n = 6.28%) were self-employed, and seven (n = 7.33 %) were unemployed. A majority of participants (n = 16.76 %) reported having heard of condoms; however, only two participants (9.5 %) reported ever having used condoms. For the main demographic characteristics please see Table 1.

**Table 1:** Demographic characteristics.

No.	pseudonyms	Age	Educational status	Marital Status
1.	H	26	High school	Single
2.	F	21	University student	Single
3.	N	24	Middle school	Single
4.	K	29	High school	Single
5.	S	28	High school	Single
6.	J	31	Bachelor	Single
7.	Y	35	Middle school	Married
8.	D	22	High school	Married
9.	N	26	Middle school	Single
10.	S	25	University student	Single
11.	F	25	High school	Single
12.	X	31	Bachelor	Single
13.	M	26	Middle school	Single
14.	A	23	High school	Single
15.	S	26	High school	Single
16.	L	24	Middle school	Single
17.	AA	29	Middle school	Single
18.	X	35	High school	Married
19.	H	18	University student	Single
20.	T	23	Middle school	Single
21.	H	27	University student	Single

**Fig. 1:**
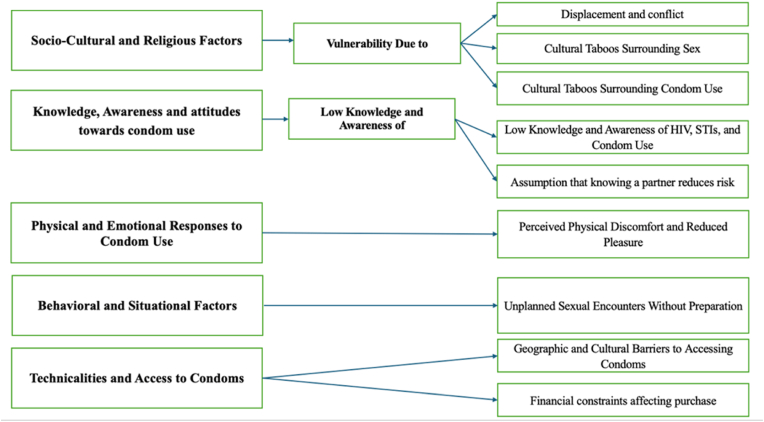
**Analytical framework showing the five thematic themes and their corresponding sub-codes/categories.** The arrows illustrate the relationship between the broader thematic barrier and its specific manifestations that contribute to vulnerability and barriers to condom use.

The codes and themes that emerged from the data transcripts are illustrated in Fig. 1. Five thematic themes were identified: 1) socio-cultural and religious; 2) knowledge, awareness and attitudes towards condom use; 3) physical and emotional responses to condom use; 4) behavioral and situational factors; and 5) technical factors influencing condom use. The arrows within the figure denote the linkage between the primary thematic themes (left) and their constituent sub-categories/codes (right). These themes capture barriers and challenges, such as vulnerability due to displacement, actors influencing condom use included limited access to sexual health education, lower awareness of HIV and STIs, perceived physical discomfort, and financial constraints. Each of these factors underscores both individual and contextual barriers to condom use. (See Fig. 1). Participant quotes have been lightly edited for clarity, preserving the original meaning.

### 3.2 Codes and themes

In exploring the factors influencing attitudes and behaviors towards condom use, particularly among vulnerable and displaced populations, it is essential to categorize the diverse and interconnected factors shaping these dynamics. The following thematic themes were developed based on a thorough qualitative analysis, providing a structured framework to understand the complexities of sexual health practices and barriers within this context.

The codes are organized into five primary thematic themes, each addressing a distinct yet interrelated aspect of the issue. These themes highlight the socio-cultural, psychological, and practical barriers faced by individuals, alongside situational and systemic challenges. By identifying these themes, the study aims to shed light on key areas for targeted interventions and policy improvements.

1. Socio-Cultural and Religious Factors

This category encapsulates the influence of displacement, cultural norms, and religious beliefs on perceptions and practices related to condom use. It examines how societal taboos surrounding sex, limited sexual health education, and fear of social judgment hinder open discussions and adoption of preventive measures.

⁃ Vulnerability Due to Displacement and Cultural Taboos Surrounding Sex and Condom Use

Young males displaced due to conflict in Libya face significant vulnerability when it comes to sexual health. Displacement forces them into environments where survival finding food, water, and shelter becomes the primary focus, pushing health protection, including condom use, to the background. In addition to the challenges of displacement, cultural taboos surrounding sex and condom use further exacerbate this vulnerability. In Libya's traditional communities, discussions about sex, particularly among unmarried individuals, are considered inappropriate, making it difficult for young men to seek information about sexual health or access condoms. The taboo extends to even mentioning condoms, as it can lead to social stigma and labeling, which discourages individuals from prioritizing their own sexual health.

"*Living in these camps, everything feels uncertain. You're focused on finding food or a safe place to sleep, so things like condoms don’t even cross your mind … It’s not that we don’t care; it’s just that survival is more immediate.*" (Age 26, H)

“*Islam does not allow any form of intimacy before marriage, so condoms are irrelevant in that context … … … Sex before marriage is “haram” forbidden, and using condoms for sex makes it even worse in the eyes of the community.*” (Age 26, H)

"*In our culture, talking about sex is already difficult, but mentioning condoms is even harder. It feels like you’re breaking some unspoken rule. People see you differently if you bring it up, and no one wants that stigma.*" (Age 21, F)

"*If someone knows you use condoms, they think you’re up to no good. It’s not fair, but that’s just how people think … in our society they judge even what you eat and what you wear.*" (Age 22, D)

⁃ Lack of Sexual Health Education and Fear of Social Judgment

A lack of sexual health education, both at home and in schools, leaves young displaced men ill-prepared to make informed decisions about their sexual health. Many grow up without proper guidance on safe sex practices, including condom use, and IDP communities often lack formal health services or programs that could provide this essential information. Attempting to purchase or use condoms can lead to ridicule or harsh judgment, with young men being labeled as morally loose or irresponsible. This social stigma creates a significant barrier, preventing them from seeking protection and making informed sexual health decisions.

"*We never talked about this at home. Even at school, nothing was mentioned about how to protect yourself. I had to learn things the hard way, through friends who also didn’t really know much.*" (Age 24, N)

"*No one comes here to talk about health or condoms. If you don’t know where to go for help, you just stay in the dark.*" (Age 23, T)

2. Knowledge, Awareness and attitudes towards condom use

This theme focuses on the knowledge gaps and misconceptions about HIV, sexually transmitted infections (STIs), and the protective role of condoms. It also explores the false sense of security derived from personal relationships and how this influences risk perception and decision-making.

⁃ Low Knowledge and Awareness of HIV, STIs, and Condom Use

A common belief among young men is that if they trust their partner or if no visible symptoms are present, they do not need to use condoms. This misunderstanding increases the risk of infection, particularly in IDP settings where health resources are limited. Moreover, many young men do not recognize the signs or symptoms of STIs, making it difficult to seek medical attention early. In addition to limited knowledge about HIV and STIs, many young IDPs have insufficient education on proper condom use. This leads to incorrect use, reducing their effectiveness and fostering distrust in their reliability. Without proper guidance on condom usage, some young men abandon their use altogether, feeling uncertain about their efficacy. Furthermore, limited access to condoms is compounded by the fact that many young men do not know where to obtain them or feel too embarrassed to ask. In Libya's IDP settings, there is little to no promotion or visibility of condom availability, making it even harder for young people to access protection discreetly. Many young men perceive condoms negatively, associating them with reduced pleasure or viewing them as unnecessary in certain situations. This attitude prevents consistent condom use, especially in contexts where condomless sex activities may be normalized.

"*We’ve heard of HIV, but a lot of us don’t know exactly how it’s transmitted. … You could have an infection and not know it.*" (Age 22, D)

"*Even when I’ve had condoms, I wasn’t sure how to use them the right way. If you don’t know, it’s easy to mess it up …. Some people just give up on using them because they don’t know if it’ll even work.*" (Age 21, F)

"*I wouldn’t even know where to get a condom around here. It’s not like they advertise it, and you don’t want to ask around. You are in Libya, and if you look around, there are no signs or promotion about condoms.*" (Age 24, N)

"*There’s this idea that condoms ruin the moment. A lot of guys don’t want to use them because they think it’s not as good.*" (Age 29, K)

⁃ Assumption that knowing a partner reduces risk

Trust in a partner often leads young men to believe that condoms are unnecessary. As relationships progress, discussions about condom wane. Many young men avoid discussing condom use with their partners out of fear of causing conflict. In conservative cultures, discussing sexual health is often seen as taboo; fear of how a partner might react to the suggestion of using condoms often keeps young men from insisting on their use.

"*Once you’re with someone for a while, you stop thinking about condoms. You think you know them well enough.*" (Age 28, S)

"*In the beginning, you might be more careful, but over time, you get comfortable. You don’t even think about using condoms anymore.*" (Age 31, J)

"*Bringing up condoms can feel like you’re accusing your partner of something. It’s easier to avoid the conversation altogether …..It’s embarrassing to talk about condoms. You don’t want to sound like you’re too eager, so you stay quiet … ….. What if they think you don’t trust them?*" (Age 35, Y)

3. Physical and Emotional Responses to Condom Use

This category delves into participants’ reported physical and emotional experiences with condom use, such as discomfort and concerns about reduced sexual pleasure.

⁃ Perceived Physical Discomfort and Reduced Pleasure

For many young men, the physical experience of using condoms can be a deterrent, as reports of discomfort are common. Complaints range from condoms feeling too tight, causing irritation, or even leading to a sense that they diminish sexual pleasure. The sensation of tightness or irritation might be due to improper sizing, incorrect application, or even allergies to latex, but in many cases, young men do not have the knowledge or resources to address these issues. Instead of seeking alternatives they often just avoid condoms altogether. This discomfort is not just physical but emotional as well. The belief that condoms reduce sexual pleasure can lead to reluctance in using them, as many young men prioritize the immediate gratification of pleasure over long-term health protection.

"*Some guys say it just doesn’t feel right when they use condoms. They complain that it’s too tight, or it irritates their skin … We hear it all the time from friends and even see it in the media—people saying condoms just kill the mood.*" (Age 22, D)

4. Behavioral and Situational Factors

This theme addresses situational variables, such as the prevalence of unplanned sexual encounters, which often lead to unpreparedness and lack of condom use. It explores the behavioral patterns that arise in these scenarios.

⁃ Unplanned Sexual Encounters Without Preparation

Unplanned or spontaneous sexual encounters often leave young men unprepared, both mentally and physically, to practice safe sex. The unpredictability of these situations bears the risk of simply not having condoms available at the moment when it is most needed. This lack of preparation is not only due to the spontaneous nature of the encounters but also because carrying condoms is sometimes seen as unnecessary or inconvenient. Additionally, some young men may feel that having condoms readily available implies they are planning for sex, which could be socially frowned upon, especially in more conservative environments. As a result, these situations increase the likelihood of condomless sex activities, as the urgency or excitement of the moment often overrides the consideration for protection. Even if the intention to use condoms exists, the lack of immediate access during spontaneous encounters leads to increased instances of condomless intercourse.

"*Things happen unexpectedly, and when you’re caught off guard, you’re not thinking about protection ….. There’s no planning involved when it happens unexpectedly. You don’t carry condoms around just in case.*" (Age 26, C)

"*When things happen quickly, you might not even have a condom around. Even if you want to use one, there’s no way to get one right then and there.*" (Age 25, G)

"*I have no idea where I can check myself, and if you’re not using condoms one time, I believe it's not a big deal.*" (Age 22, D)

5. Barriers to Access to Condoms
  ⁃ Geographic and Cultural Barriers to Accessing Condoms

Young men in IDP settings face barriers to obtaining condoms, both geographically and culturally. In many IDP camps free or affordable condoms are not available. Geographic isolation, combined with poor infrastructure, makes it difficult for young men to obtain condoms even when they are aware of the need for protection.

Additionally, cultural restrictions and social stigma often discourage young men from purchasing them. In culturally sensitive to sexual norms, buying condoms can be seen as taboo, and the fear of being judged by store owners or recognized by someone they know creates discomfort.

*"We’re far from any place where you could get condoms … There’s nothing in these camps when it comes to sexual health. If you want condoms, you’d have to go to the city."* (Age 25, R)

*"It’s hard to buy condoms because you don’t want people to see you. You worry about what they’ll think or say … Going into a pharmacy to buy condoms can feel like everyone’s watching you. You don’t want to be seen as the guy buying condoms, so you just don’t do it."* (Age 31, X)

⁃ Financial constraints affecting purchase

Financial constraints significantly affect the ability to purchase condoms, especially in post-conflict settings like Libya. Many young people in these communities are struggling with financial instability due to the disruption of their lives, loss of employment opportunities, and displacement from their homes. With limited income, basic needs such as food, shelter, and healthcare take priority over other purchases. For many IDPs, the focus is on immediate survival and family responsibilities, pushing the need for condoms lower down the list of priorities. Additionally, in areas affected by conflict, access to affordable healthcare and sexual health resources is limited. Pharmacies might not stock condoms regularly, or they may be more expensive than in more stable areas. The overall economic hardships further exacerbate this problem, as IDPs typically lack access to subsidized or free sexual health resources, like condoms, which might be available in more stable regions.

"*I’ve heard about condoms, but I don’t know much about how to use them or why they’re so important. Plus, even if I did, I can’t really afford to spend money on something I don’t fully understand or prioritize. Right now, I’m just trying to survive.*" (Age 26, M)

"*Most of us don’t have extra money to spend on condoms. We’re focused on getting by with food and family needs, and condoms feel like a luxury we can’t afford.*" (Age 23, A)

"*We don’t get condoms for free around here, and with no steady income, it’s not something we can always afford. Sometimes, we just take the risk because there’s no other option.*" (Age 24, L)

## 4 Discussion

Our study reveals significant gaps in sexual health knowledge, attitudes, and behaviors toward condom use among young, male IDPs in Libya. Only a small proportion of participants mentioned having used condoms at least once. Key barriers included lack of sex education, restrictive cultural norms, stigma associated with condom use, and limited access to condoms. These findings resonate with previous studies on the sexual health of displaced populations and migrants, especially in conflict-affected regions, where socio-economic and geographic factors significantly impact health-seeking behaviors and limit access to sexual and reproductive health (SRH) services [21,22].

There is evidence that IDPs experience a disproportionate burden of sexual and reproductive health problems compared to non-IDPs which can lead to higher rates of unintended pregnancies, adolescent pregnancies and STIs [23,24]. Displaced populations often face disrupted access to education, including sex education, which leads to a gap in knowledge about STIs and preventive methods like condoms or more recently preexposure prophylaxis (PrEP). Our study found that most young men did not fully understand how STIs and HIV are transmitted, nor were they adequately informed about the correct use of condoms or other prevention methods. Participants reported that their knowledge about sexual health came mainly from peers, which is often inaccurate or incomplete. In a recent cross-sectional study from Morocco assessing prevalence and determinants of intercourse condomless among migrants and refugees in Morocco, being homeless, having difficulty obtaining condoms, and only having a basic education were all risk factors for these sexual behaviors [25]. Cultural and religious beliefs play a crucial role in shaping sexual health practices, particularly in conservative societies like Libya [26,27]. Our study participants described how societal and religious norms created barriers to discussing or using condoms. This finding aligns with other research from conflict-affected Muslim-majority regions, where cultural taboos make open discussions about sexual health and condom use challenging [28]. The influence of religion in the region is strong, and interventions should be developed within these frameworks [26]. Leverage on religious teachings to promote the use of condoms as a means of safeguarding of one's health and that of others would be useful.

Geographic isolation and economic challenges were also highlighted as major barriers to condom use in our study. Many participants described how their displacement to IDP camps limited their access to condoms, both geographically and financially. This is consistent with global reports from humanitarian organizations that emphasize the scarcity of SRH resources in conflict zones [[29], [30]]. Thus, targeted interventions are needed to improve access to sexual and reproductive health (SRH) resources in conflict-affected areas.

The participants in our study also frequently reported misconceptions, such as believing that they could determine whether a partner had an STI based on appearance or that trust in a partner eliminated the need for condoms. While it is acknowledged that the risks associated with condomless sex vary significantly based on relational context (e.g., fidelity) and reproductive goals, these misconceptions dangerously oversimplify risk in a high-vulnerability setting. These misconceptions have been reported in other studies, where knowledge about SRH is limited or based on myths 30). In our study, these misconceptions contributed to condomless sex activities, such as engaging in condomless sex.

The findings from this study have significant policy implications for humanitarian and public health organizations working in conflict zones like Libya. There is an urgent need for culturally sensitive interventions that address the specific barriers young men face in accessing and using condoms. This includes providing comprehensive sex education within IDP camps, ensuring the availability of free or affordable condoms, and combating the stigma associated with condom use. World Health Organization (WHO) has advocated for the integration of SRH services into broader humanitarian aid programs, particularly in conflict-affected regions [31]. Our study supports this recommendation, emphasizing the importance of SRH interventions tailored to the unique needs of IDPs. These may include **mobile SRH clinics within IDP camps, culturally sensitive condom distribution programs, peer-led education sessions, and engagement with religious and community leaders to address stigma and misconceptions**. Additionally, the World Health Organization recommends community-based education programs that involve religious and community leaders to help reduce stigma and increase the acceptability of condoms in conservative societies.

### 4.1 Study limitations

With a small sample size of 21 participants, all of whom were heterosexual men from IDP camps in Tripoli, the results cannot be broadly applied to other regions or populations, especially those in rural areas with different access to resources. The study also does not include the perspectives of women, LGBTQ + individuals, or other marginalized groups, which limits its scope in understanding these populations.

The reliance on self-reported data introduces potential biases, as participants might not fully recall or accurately report their behaviors and knowledge. Furthermore, because the study was conducted at a single point in time, it does not capture changes in attitudes or behaviors over time. Finally, the decision not to audio-record interviews due to confidentiality concerns may have affected the richness of the data, as important non-verbal cues and subtle details could not be fully captured. These limitations suggest specific avenues for future research, including: 1) conducting mixed-methods studies to quantify the prevalence of identified barriers; 2) expanding research to include the perspectives of female IDPs and local healthcare providers to achieve triangulation; and 3) exploring the feasibility of culturally sensitive mobile SRH clinics through implementation science research in IDP settings.

### 4.2 Strengths

The study offers several key strengths that enhance its value in understanding sexual health among young displaced men in Libya. It focuses on a vulnerable and understudied group, providing important insights into the specific barriers they face regarding condom use in a post-conflict setting. The qualitative approach allowed for in-depth exploration of participants' attitudes and experiences, generating rich data that highlights key factors such as cultural taboos, stigma, and economic barriers. Conducting interviews in the participants' native language ensured cultural relevance and accurate data collection. Additionally, the study provides actionable insights for developing culturally sensitive interventions to improve sexual health outcomes in conflict-affected regions, making a significant contribution to the public health field.

### 4.3 Conclusion

In conclusion, this study highlights the complex interplay of geographic, cultural, and economic factors that hinder condom use among young displaced men in Libya. The findings underscore the need for targeted interventions that address not only the lack of access to condoms but also the cultural and social barriers that prevent their use. By addressing these multifaceted challenges, public health organizations can significantly improve sexual health outcomes for displaced populations in conflict zones.

## Consent for publication

All coauthors provide consent for publication.

## Availability of data and materials

All interviews are documented and available.

## Ethical statement

The study received clearance and approval to conduct the research through the Ethical Regulatory Authority of the Department of Health, Libya. And the Review Committee for Research Involving Human Research Subjects, Health Science Group Chulalongkorn University. Bangkok, Thailand under COA No.083/66, and from the Ethical Regulatory Authority of the Department of Health, Libya. Potential participants were informed about the nature and purpose of the study when they were invited to participate. We obtained consent from all participants.

## Funding

This study received no specific funding from any agency in the public, commercial, or not-for-profit sectors.

## Declaration of competing interest

The authors declare that they have no known competing financial interests or personal relationships that could have appeared to influence the work reported in this paper.
